# Early Influences

**Published:** 1875-12

**Authors:** 


### EARLY INFLUENCES.

There can be no greater blessing than to be born in the light and air of a cheerful, loving home. It not only insures a happy childhood—if there is health and a good constitution—but it almost makes sure a virtuous and happy manhood, and a fresh young heart in old age. We think it every parent's duty to try to make their children's childhood full of love and of childhood's proper joyousness; and we never see children destitute of joy through the poverty, faulty tempers, or wrong notions of their parents, without a heartache. Not that all the appliances which wealth can buy are necessary to the free and happy unfolding of childhood in body, mind, or heart—quite otherwise; but children must at least have love inside the house, and fresh air and good play, and some good companionship, outside — otherwise young life runs the greatest danger in the world of withering or growing stunted, or sour and wrong, at least prematurely old and turned inward.

